# Depression and Anxiety Among Individuals Receiving Incretin Mimetic Medications: A Saudi Cross-Sectional Study

**DOI:** 10.3390/healthcare14081049

**Published:** 2026-04-15

**Authors:** Ali M. Bahathig, Ayedh H. Alghamdi, Mohammed A. Aljaffer, Mohammed A. Alblowi, Metib S. Alotaibi, Deena N. AlNouwaiser, Asma’a M. Alshehri, Abdullah M. Alhejji, Wejdan S. Alruwaili, Ghassan A. Abuseif, Ahmad H. Almadani

**Affiliations:** 1 Department of Psychiatry, College of Medicine, King Saud University, Riyadh 11451, Saudi Arabiaaayedh@ksu.edu.sa (A.H.A.);; 2 Department of Psychiatry, King Saud University Medical City, King Saud University, Riyadh 11451, Saudi Arabiagabuseif@ksu.edu.sa (G.A.A.); 3 University Diabetes Center, King Saud University Medical City, King Saud University, Riyadh 11451, Saudi Arabia; 4 Department of Psychiatry, King Faisal Specialist Hospital, Riyadh 11451, Saudi Arabia

**Keywords:** anxiety, depression, GLP-1 receptor agonists, incretin mimetics, Saudi Arabia

## Abstract

Background: Depression and anxiety are prevalent mental health disorders that substantially impact quality of life. The association of incretin mimetics, including glucagon-like peptide-1 (GLP-1) receptor agonists, with symptoms of depression and anxiety remain underexplored in Saudi Arabia. This study was conducted to assess the association between GLP-1 receptor agonist use and symptoms of depression and anxiety and to identify related factors. Methods: A cross-sectional study using convenience sampling was conducted among adults (≥18 years) treated with GLP-1 receptor agonists at King Khalid University Hospital (KKUH) in Riyadh, Saudi Arabia. Data were collected using a questionnaire developed by the research team, in addition to the Arabic versions of the Patient Health Questionnaire-9 (PHQ-9) and the Generalized Anxiety Disorder-7 (GAD-7). Results: A total of 235 participants were included, of whom 48.5% used GLP-1 receptor agonists for both glycemic control and weight loss. Only 31.9% had undergone psychiatric evaluation prior to initiating therapy, and 14.9% had a diagnosed psychiatric disorder. The mean anxiety score (GAD-7) was 4.82 ± 5, and the mean depression score (PHQ-9) was 6.13 ± 4.95. Multivariable analysis showed that higher odds of more severe depression were associated with using diabetes medications for weight loss in addition to diabetes treatment, a history of psychiatric disorders, and holding a bachelor’s degree. Exercising for 101–150 min per week was associated with lower odds of depression. Regarding anxiety, participants who exercised 101–150 min per week had significantly lower odds of anxiety compared with those who did not exercise, while a history of psychiatric disorders was associated with higher odds of more severe anxiety. Conclusions: This study’s findings highlight the importance of integrating both routine psychiatric screening and follow-up into diabetes and obesity management to enhance both psychological well-being and metabolic outcomes. They also reflect the benefit of physical activity for mental health, emphasizing the need to encourage exercise among individuals with diabetes or obesity.

## 1. Introduction

Depression is a common mental disorder that significantly impairs individuals’ daily functioning [1]. Globally, the lifetime prevalence of depression is estimated to range between 10% and 15%, with higher rates observed among women and individuals with chronic medical conditions [2]. Anxiety disorders, by contrast, are characterized by excessive fear and worry [1], affecting multiple domains of daily life [1]. Epidemiological evidence suggests that up to 33.7% of the global population may experience an anxiety disorder at some point during their lifetime [3]. Regionally, countries in the Middle East and North Africa report depression prevalence rates of 9.2% among females and 5.6% among males, while the prevalence of anxiety disorders is estimated at 8.1% for females and 4.1% for males [4]. In Saudi Arabia, nationwide estimates indicate that the prevalence rates of general anxiety disorder and major depressive disorder are 12.4% and 12.7%, respectively [5].

Obesity is a common and significant health problem worldwide [6]. According to a systematic review, obesity affects a substantial portion of the Saudi population, with rates spanning 20–39% in adults, and type II diabetes mellitus is the second most common obesity-related comorbidity (60.7%) [7]. Further, based on a systematic review and meta-analysis, the pooled national prevalence of prediabetes among Saudi adults is estimated at 24.1% [8]. A Saudi-based study documented substantial rates of mental health conditions among individuals with type II diabetes, with 12.4% reporting anxiety and 23.5% reporting depression [9].

Glucagon-like peptide-1 (GLP-1) is a hormone that regulates appetite, stimulates glucose-dependent insulin secretion, and exerts cardioprotective and neuroprotective effects, including improving memory, neurogenesis, and neurotransmission [10,11]. Use of GLP-1 receptor agonists (RAs) for weight management in non-diabetic individuals surged by over 700% in the United States between 2019 and 2023 [12], while in Saudia Arabia, research on their long-term safety and expanding applications continues [13]. Use of tirzepatide, a dual glucose-dependent insulinotropic polypeptide (GIP)/GLP-1 receptor co-agonist, has been demonstrated to reduce HbA1c by 1.24–2.58% and result in weight loss of 5.4–11.7 kg in clinical trials [14], with preliminary findings also suggesting that it offers psychiatric benefits in conditions such as bipolar disorder [15].

Further, studies conducted on GLP-1 RAs, including randomized controlled trials (RCTs), indicate that GLP-1 RAs are broadly well-tolerated, with gastrointestinal symptoms being the most common adverse effect [16,17,18,19,20]. Other side effects have also been reported [17,18,19]. In terms of contraindications, thyroid-related cancers are among the most significant [17]. Regarding GLP-1 RAs’ psychiatric effects, the U.S. Food and Drug Administration (FDA) originally highlighted a potential risk of suicidal ideation [20], but after reviewing available evidence, the found no increased risk of suicidality with GLP-1 RAs and removed the prior requirement of such warnings on their labeling [20]. Furthermore, some studies reported significant reductions in depression scores with GLP-1 RAs compared with controls [21,22]. Conversely, an analysis of associated psychiatric adverse events indicated that depression accounted for 50.3% of reported cases, anxiety for 38.7%, and suicidal ideation for 19.6% [23].

Although there are international guidelines for the treatment of obesity, including for newer interventions such as GLP-1 RAs [24,25], no formal national obesity guideline exists in Saudi Arabia. However, one study discussed clinical practice for the management of overweight and obesity in the Saudi context [26]; this study suggested that GLP-1 RAs can be administered to adults with obesity (body mass index (BMI) ≥ 30 kg/m^2^) or overweight (BMI ≥ 27 kg/m^2^) with adiposity-related complications, in conjunction with lifestyle and behavioral changes [26]. It also recommended GLP-1-based therapy for patients with DM-II and overweight/obesity, especially those presenting with cardiovascular risk factors [26]. The study further suggested that all patients with overweight or obesity should undergo routine mental health screening at baseline and during follow-up, noting that mental health screening is not a GLP-1-specific mandatory clinical prerequisite to initiate GLP-1 RA treatment [26].

Given the rapid increase in GLP-1 RA use [12] and the growing body of evidence suggesting their potential mental health effects [21,22], there is a pressing need to examine the psychological outcomes of patients receiving these medications in Saudi Arabia. To our knowledge, this study is one of the first studies to evaluate the associations of use of GLP-1 RAs with psychiatric symptoms within a Saudi population, thus addressing a notable gap in the literature on this region. Specifically, this study was conducted to assess the prevalence of depression and anxiety among patients prescribed GLP-1 RA medications through a descriptive characterization of the mental health profile of this patient population, with the aim of better understanding the associations with psychiatric symptoms. The findings can inform clinical guidelines on mental health screening and monitoring of patients on GLP-1 RAs. Furthermore, this study can serve as a foundation for future research investigating the neuropsychiatric mechanisms associated with these agents. The findings could enrich the literature, not only within the Saudi context but also internationally, allowing for a comparison between Saudi Arabia and other locations.

## 2. Materials and Methods

### 2.1. Study Design, Setting, and Participants

This cross-sectional study was conducted in Riyadh, Saudi Arabia. Data were collected from patients attending the diabetic center at King Khalid University Hospital (KKUH). The target population consisted of adult patients diagnosed with diabetes and/or obesity who were receiving GLP-1 RA medications at the time of the study. The inclusion criteria were adults aged 18 years and older who had been prescribed GLP-1 RAs or tirzepatide for the management of diabetes and/or obesity, while the exclusion criteria were individuals younger than 18 years and those with communication barriers that could interfere with participation. The required sample size was calculated at a 95% confidence level and a 5% margin of error using the Raosoft calculator (https://www.calculator.net/sample-size-calculator.html) (accessed on 25 January 2025). Based on the target population size of 437 obtained from the information technology (IT) department, and accounting for a 15% increase to account for nonresponders or incomplete responses, the total estimated sample size was 236 participants.

Data were collected remotely using a digital version of the study tool developed on SurveyMonkey.com (accessed on 10 February 2025). The research team obtained a list of eligible patients’ medical record numbers and corresponding contact information from the hospital’s IT department. Eligible participants were then contacted virtually, invited to participate in the study, and provided with information regarding the nature of the study and their rights as participants, including that participation was voluntary. They were then given access to the online study tool for data collection. A few patients in the sample, around ten, required assistance from a family member, namely a first-degree relative, to complete the survey due to limited familiarity with online surveys; however, all responses were provided directly by the participants.

### 2.2. Study Instruments

Data for this study were collected using an electronic survey. The survey comprised a questionnaire developed by the research team to collect sociodemographic and clinical information, in addition to two Arabic-validated screening tools, the Patient Health Questionnaire-9 (PHQ-9) and the Generalized Anxiety Disorder-7 (GAD-7), which were used to assess symptoms of depression and anxiety, respectively.

The PHQ-9 is a widely used instrument for screening, diagnosing, monitoring, and assessing the severity of depression. It consists of nine multiple-choice items, each scored from 0 to 3, yielding a total score ranging from 0 to 27. Higher scores indicate greater depressive symptom severity. A score greater than 10 has demonstrated a sensitivity and specificity of 88% for detecting major depression [27]. Depression severity is categorized as mild (5–9), moderate (10–14), moderately severe (15–19), and severe (20–27). The PHQ-9 has demonstrated strong psychometric properties, including excellent test–retest reliability [27] and high internal consistency, with Cronbach’s α values of 0.89 in the PHQ Primary Care Study and α = 0.86 in the PHQ Ob-Gyn Study [27,28]. The Arabic version of the PHQ-9, translated and validated by Alhadi et al., also showed internal consistency, with a Cronbach α of 0.857 and inter-item correlations ranging from 0.177 to 0.648 [29].

The GAD-7 is a validated tool used to screen for GAD and to assess symptom severity in both clinical and research settings. It includes seven items, each scored from 0 to 3, resulting in a total score ranging from 0 to 21, with higher scores indicating greater anxiety severity. Anxiety severity is categorized as minimal (0–4), mild (5–9), moderate (10–14), and severe (15–21). The GAD-7 has demonstrated excellent internal consistency, with a Cronbach α of 0.93, and strong test–retest reliability, with an intraclass correlation of 0.83 [30]. The Arabic version, translated and validated by Alhadi et al., also showed acceptable internal consistency (Cronbach’s α = 0.763), with inter-item correlations ranging from 0.204 to 0.426 [29]. This study utilized the Arabic versions of the PHQ-9 and GAD-7.

### 2.3. Ethical Considerations

The survey started with an introductory statement outlining the study title and objectives. The statement emphasized that participation was entirely voluntary and included the name and contact information of the principal investigator in case the participants had any inquiries. The participants were assured that collected data would remain strictly confidential. Ethical approval for the study was obtained from the Institutional Review Board of the College of Medicine at King Saud University (Research Project Number: E-25-9502). Informed consent was obtained electronically; participants indicated their agreement to participate by clicking “next” to proceed with the survey.

### 2.4. Statistical Analysis

Statistical analysis was performed with the Statistical Package for the Social Sciences (SPSS) version 28 (IBM Co., Armonk, NY, USA). Numerical data were presented as the means and standard deviations (SDs), while categorical data were presented as the frequency and percentage. Ordinal logistic regression analyses were performed to assess factors associated with depression and anxiety levels. A two tailed *p*-value < 0.05 was considered statistically significant.

Variables for inclusion in the ordinal logistic regression models were selected using a two-step approach. All candidate variables were first entered into a univariate ordinal logistic regression model. Variables with *p* < 0.20 at the univariate level, along with a priori clinically justified variables (age, sex, BMI, DM diagnosis, and GLP-1 duration), were retained for entry into the multivariable model. Psychosocial modifiers, including educational level, monthly income, and exercise duration, were included based on established evidence linking socioeconomic factors and physical activity to mental health in populations with chronic diseases [31,32,33]. Although the inclusion of non-significant univariate variables might introduce noise, retaining them enabled adjustment for potential confounding and a more complete estimation of independent predictors.

The proportional odds assumption for ordinal logistic regression was assessed using the Brant test and was not violated in the final models. Multicollinearity was assessed using variance inflation factors (VIFs); all VIF values were <3, indicating no problematic collinearity.

Both the validated and culturally adapted versions of the GAD-7 and PHQ-9 demonstrated good to excellent internal consistency in this sample. The GAD-7 showed excellent reliability (Cronbach’s α = 0.926), and the PHQ-9 showed good reliability (Cronbach’s α = 0.868), confirming the suitability of both instruments for use in this population.

## 3. Results

A total of 235 respondents receiving (GLP-1) RA medications were included in our study, more than half (52.3%) of whom were 46 years old or older. The mean BMI was 34.09 ± 8.6 kg/m^2^, with a female predominance (59.1%). Married participants accounted for the largest group (63.4%), and the predominant monthly income was <10,000 Saudi Riyals (55.7%). The highest educational level was reported to be a Bachelor’s degree, which 48.1% had obtained. Moreover, 39.1% of participants reported exercising for <50 min a week. The most common comorbidity was hypertension (HTN), reported by 34% of participants. The prevalence of diabetes (DM) was 60%; most participants with diabetes (55.3%) have had their diagnosis for over 10 years. Further, the majority of participants had not been hospitalized or admitted to the emergency department for DM complications in the past year (74.5% and 69.5%, respectively). The vast majority (95%) were taking DM medications, with 25.1% only taking Metformin. GLP-1 RAs such as Saxenda, Ozempic, and Mounjaro had been used for less than a year among 52.3% of participants, and 48.5% reported using such medications for weight loss in addition to treating DM. Slightly less than one-third of participants (31.9%) were evaluated by psychiatrists before using GLP-1 medications, and 14.9% were diagnosed with psychiatric disorders, with mood and anxiety disorders diagnosed in 54.3% and 48.6%, respectively, of whom 85.7% were taking antidepressants/anti-anxiety medications. The majority (74.3%) were diagnosed with a psychiatric illness before starting a GLP-1 RA medications, and 12.8% reported having first-degree relatives with a psychiatric illness [Table 1].

Responses to the GAD-7 questionnaire are descriptively depicted in Table 2, revealing that the problems that participants experienced most over the past two weeks were primarily trouble relaxing (mean score: 0.89 ± 0.93) and becoming easily annoyed or irritable (0.85 ± 0.88), followed by feeling nervous, anxious, or on edge (0.83 ± 0.9) and worrying too much about different things (0.74 ± 0.89), although 42.1%, 41.3%, 43.4%, and 49.8%, respectively, of participants reported not experiencing these problems at all. Overall, no, mild, moderate, and severe anxiety were present in 57.4%, 25.5%, 12.3%, and 4.7% of participants, respectively, with a mean total score of 4.82 ± 5. Importantly, these problems did not impact 56.6% of patients’ ability to work, take care of things at home, or get along with other people, while 31.9% of patients reported that their symptoms made these tasks somewhat difficult.

As for the PHQ-9 questionnaire, the highest scores were obtained for poor appetite or overeating (mean 1.23 ± 0.93, with 40% suffering for several days); feeling tired or having little energy (1.17 ± 0.93, with 43.4% suffering for several days); trouble falling or staying asleep, or sleeping too much (1.05 ± 0.96, with 34.9% not suffering at all and 34.5% suffering for several days); feeling down, depressed, or hopeless (0.71 ± 0.75, with 45.5% not suffering at all and 40% suffering for several days); and little interest or pleasure in doing things (0.6 ± 0.77, with 55.3% not suffering at all and 31.9% suffering for several days). Accordingly, the patients were categorized into five groups, in which 43.8% had no depression, 31.9% had mild depression, 16.2% had moderate depression, 7.2% had moderate-to-severe depression and 0.9% had severe depression, with a mean total score of 6.13 ± 4.95. Moreover, 13.2% were diagnosed with a major depressive disorder. Their symptoms did not make it difficult at all for 57% of patients to work, take care of things at home, or get along with other people, while they made these tasks somewhat difficult for 32.8% [Table 3].

In the univariate ordinal logistic regression analysis, participants who exercised for 101–150 and >150 min a week showed significantly lower odds of being more anxious than those who did not exercise at all, with odds ratios (ORs) (95%CI) of 0.32 (0.11 to 0.94, *p* = 0.039) and 0.26 (0.08 to 0.84, *p* = 0.025), respectively. Participants with a history of using DM medications for weight loss in addition to treating DM, those who were evaluated by a psychiatrist before initiating a GLP-1 medication, such as Saxenda, Ozempic, or Mounjaro, those who were diagnosed with any psychiatric disorder, and those with a first-degree relative (parents, siblings, or children) with a psychiatric illness showed significantly higher odds of experiencing more severe anxiety than other participants, with ORs (95%CI) of 1.73 (1.05 to 2.86, *p* = 0.032), 1.84 (1.09 to 3.09, *p* = 0.022), 9 (4.51 to 17.96, *p* < 0.001), and 3.3 (1.63 to 6.65, *p* = 0.001), respectively.

In the multivariable regression model, participants who exercised 101–150 min a week showed significantly lower odds of anxiety than those who did not exercise at all (OR = 0.25, 95%CI: 0.07 to 0.9, *p* = 0.035). Also, participants with a history of psychiatric disorders showed significantly higher odds of experiencing anxiety than other participants (OR = 9.17, 95%CI: 4.08 to 20.64, *p* < 0.001) [Table 4].

In the univariate ordinal logistic regression analysis, a higher BMI was significantly associated with more severe depression (OR = 1.03, 95%CI: 1 to 1.06, *p* = 0.025). Hypertensive patients showed significantly higher odds of being more depressed than non-hypertensive patients (OR = 1.75, 95%CI: 1.06 to 2.88, *p* = 0.028). Moreover, participants with a history of using DM medications for weight loss in addition to treating DM, those who were evaluated by a psychiatrist before initiating a GLP-1 medication, such as Saxenda, Ozempic, or Mounjaro, those who were diagnosed with any psychiatric disorder, and those with a first-degree relative (parents, siblings, or children) with a psychiatric illness showed significantly higher odds of more severe depression than others, with ORs (95%CI) of 2.36 (1.45 to 3.83, *p* = 0.001), 1.78 (1.07 to 2.96, *p* = 0.026), 9.04 (4.36 to 18.74, *p* < 0.001), and 3.08 (1.53 to 6.23, *p* = 0.002), respectively.

After adjusting for the included factors, participants with a Bachelor’s degree or a Master’s or PhD degree showed significantly higher odds of experiencing more severe depression than the uneducated participants (OR = 4.7, 95%CI: 1.03 to 21.5, *p* = 0.046; and 6.91 (1.18 to 40.44), *p* = 0.032). In comparison to participants who did not exercise at all, those who exercised 101–150 min a week showed significantly lower odds of depression (OR = 0.27, 95%CI: 0.08 to 0.88, *p* = 0.03). Also, participants with a history of using DM medications for weight loss in addition to treating DM and those who were diagnosed with a psychiatric disorder showed significantly higher odds of more severe depression than others, with ORs (95%CI) of 2.76 (1.5 to 5.1, *p* = 0.001) and 10.64 (4.51 to 25.14, *p* < 0.001), respectively [Table 5].

## 4. Discussion

This study assessed the prevalence and severity of anxiety and depression symptoms among Saudi Arabian adults using GLP-1 RAs for diabetes management or weight loss, addressing a key gap in regional mental health data for this population. These findings could inform integrated endocrine–psychiatric care strategies to enable early detection of mood and anxiety disorders and guide the development of clinical guidelines and health policies for GLP-1 RA therapy.

In this study, nearly two-thirds of adult participants receiving GLP-1 RA medications had not undergone psychiatric evaluation prior to treatment initiation, which is consistent with international evidence [34,35]. Notably, existing research on GLP-1 RAs has largely excluded individuals with significant psychiatric comorbidities and included limited systematic psychiatric assessments. As a result, the mental health effects of these medications in patients with pre-existing psychiatric conditions remain uncertain [34,35], limiting real-world neuropsychiatric safety data [36]. In addition, Saudi studies have highlighted chronic disease–mental health links: Aljadani et al. (2024) reported high rates of depression/anxiety in patients with diabetes [37], and Shamiri et al. (2023) found that >50% of heart failure patients experienced clinically significant depression and anxiety symptoms [38]. Altogether, these findings suggest a need to encourage screening GLP-1 RA recipients for mood and anxiety symptoms in metabolic clinics to provide comprehensive care and enable early risk identification.

In this study, 42.5% of participants exhibited mild or severe anxiety. This finding contrasts with international evidence summarized in a large cohort study that reported reductions in anxiety symptoms among individuals with type II diabetes who were treated with GLP-1 RAs [39]. This discrepancy may be attributable to differences in study design, population characteristics, and clinical context. Our sample reflects a real-world population with heterogeneous psychiatric histories, variable treatment durations, and differing levels of exposure to metabolic and psychosocial stressors. Additionally, limited psychiatric screening prior to GLP-1 RA initiation and potential cultural differences in symptom reporting may have contributed to the higher observed anxiety burden in our study. Another plausible explanation is that individuals with pre-existing psychological vulnerability or cumulative life stress may respond differently to GLP-1 RA therapy, particularly during treatment initiation, possibly due to interactions between central incretin signaling pathways and stress-related neural circuits [40,41,42].

In this study, 13.2% of adults receiving GLP-1 RAs met the criteria for a major depressive disorder based on PHQ-9 scores greater than 10, indicating a substantial burden of depression symptoms within this population. This finding contrasts with those of a Saudi study of adults with obesity, which reported minimal adverse psychological side effects among GLP-1 RA users [43]. The lower prevalence of depression symptoms in that study [43] may be attributable to differences in sample composition, as our cohort included a high proportion of participants with pre-existing psychiatric diagnoses and chronic metabolic diseases.

Regarding gender distribution, males represented only 39% of our sample, which may have limited the detection of sex-specific outcomes previously reported in predominantly male cohorts [44]. Future research in Saudi Arabia should investigate the longitudinal associations of GLP-1 RA therapy on mood and hormonal profiles using gender-stratified analyses. Large, multicenter Saudi studies with balanced sex representation and baseline psychiatric assessment are also needed to clarify causal relationships and identify vulnerable subgroups.

In our study, participants with a pre-existing psychiatric illness showed significantly greater anxiety/depressive symptom severity than those without. This finding aligns with an Australian study of 244 metabolic syndrome patients, in which baseline psychiatric disorders predicted post-antidiabetic weight loss-linked emotional distress [45]. From a biological perspective, dysregulation of the hypothalamic–pituitary–adrenal (HPA) axis and serotonin signaling alterations may contribute to mood disorder vulnerability [46]. A plausible hypothesis is that individuals with underlying psychiatric conditions exhibit heightened emotional reactivity to metabolic interventions due to chronic HPA axis dysregulation and disrupted serotonin pathways, sensitizing stress/reward circuits and amplifying mood fluctuations from GLP-1 receptor activation-induced hormonal/metabolic changes [22]. That being said, these mechanisms were not assessed in our study and warrant further investigation. Future Saudi research should consider examining neuroendocrine/inflammatory biomarkers as predictors of psychiatric responses and evaluate integrated behavioral–pharmacologic models. Clinically, we advise being cautious when prescribing GLP-1 RAs to patients with psychiatric comorbidities, and we recommend close mood monitoring and mental health coordination with mental health professionals.

In our study’s univariate analysis, a positive family history of psychiatric disorders was significantly associated with higher anxiety and depressive symptom scores among GLP-1 RA recipients, suggesting that inherited/familial vulnerability could influence emotional responses to treatment. This aligns with social media analyses and U.S./European registry studies showing increased anxiety, mood swings, or depression post-GLP-1 RA initiation in those with a personal/family history of mental illness [10,23,36,47]. Clinically, we suggest obtaining a detailed family psychiatric history prior to treatment to enable risk identification, closer monitoring, and timely intervention.

Among this study’s participants, the use of GLP-1 RAs, whether for glycemic control or weight loss, demonstrated a significant association with more severe depression symptoms. This finding suggests that the use of diabetes medications may be linked to an increased burden of depression symptoms. Supporting this observation, a systematic review and meta-analysis of 28 studies revealed that insulin treatment was significantly associated with a higher prevalence of depression [48]. Similarly, a large retrospective cohort study conducted in Taiwan reported an increased incidence of depression among users of GLP-1 RAs [49]. In addition, post-marketing safety reports from the U.S. FDA have identified potential depression- and anxiety-related adverse effects among individuals using GLP-1-based therapies and certain oral antidiabetic agents prescribed for diabetes and obesity [50]. Clinically, integrating routine mental health screening and counseling into metabolic and obesity care may facilitate early identification of at-risk individuals and yield benefits for both psychological well-being and metabolic outcomes.

## 5. Strengths and Limitations

This study has several strengths and limitations. One of the study’s key strengths is that it is among the few studies in Saudi Arabia to specifically examine the prevalence of depression and anxiety symptoms among patients receiving incretin mimetic medications, thereby addressing an important gap in the existing literature. The use of validated and culturally adapted Arabic versions of the PHQ-9 and GAD-7 enhances the accuracy and relevance of the assessment of depression and anxiety symptoms within the local population. In addition, the relatively large sample size strengthens the statistical power and increases the reliability of the findings.

However, several limitations should be acknowledged. First, the cross-sectional design limits our ability to draw causal inferences and prevents establishing temporal relationships between GLP-1 RA therapy and mental health outcomes. Reverse causality also cannot be excluded, as pre-existing mood/anxiety symptoms may have influenced treatment decisions or symptom reporting. Future Saudi studies should adopt a longitudinal design to clarify these temporal and causal relationships. Second, the use of convenience sampling may have introduced selection bias and limited generalizability. In addition, as the study was conducted in a single tertiary center (KKUH), the findings may not be representative of other settings in Saudi Arabia. Multi-center studies with more representative sampling are recommended. Third, data collection through phone interviews and online surveys, along with relatives assisting some participants in responding to the survey, may have introduced response bias. Using in-person interviews in future research could improve participation rates and reduce such bias. Fourth, the reliance on self-reported data may have introduced recall or social desirability biases. Incorporating clinician-administered tools or structured diagnostic interviews would improve data accuracy and reliability in future studies. Fifth, confounding by indication remains possible, as patients prescribed GLP-1 RA therapy may differ in disease severity, metabolic profile, or psychiatric vulnerability. Lastly, subgroup analyses involving smaller strata produced wide confidence intervals, indicating limited statistical power. Larger studies with standardized protocols are needed to confirm these findings.

## 6. Conclusions

Most patients in our sample did not have anxiety symptoms at the time they completed the survey; however, a larger proportion of patients exhibited depressive symptoms compared to those that did not. Nonetheless, concerning both anxiety and depression, the majority of patients exhibited symptoms of mild to moderate severity. We found that the most significant predictor for developing anxiety and depressive symptoms was a prior history of psychiatric disorders. Conversely, exercising regularly (101–150 min) was associated with lower levels of both anxiety and depression. Further, the dual use of diabetic medication for both diabetes and weight loss was correlated with higher levels of depression. No association was found between the duration of GLP-1 RA use and the development or worsening of anxiety or depression symptoms. Altogether, our findings suggest that psychosocial factors could influence psychological outcomes among patients using GLP-1 RAs, rather than psychological outcomes being related to medication exposure alone. That being said, our findings should be interpreted with caution given the cross-sectional design of the study, which precluded the assessment of cause–effect relationships. Our results support the need to implement baseline psychological evaluation and coordinated multidisciplinary team involvement, including specialists in endocrinology and psychiatry, for patients treated with GLP-1 RAs. Longitudinal Saudi cohort studies are needed to better understand the trajectory of anxiety and depression symptoms. Implementation research within Saudi clinical settings is also essential for assessing the feasibility and effectiveness of mental health screening and intervention protocols. These efforts will support the development of evidence-based practices that optimize both metabolic and psychiatric outcomes for individuals with metabolic conditions.

## Figures and Tables

**Table 1:** Participants’ socio-demographic, psychiatric, and medication data.

Item	Total Participants (*n* = 235)
Age (years)	
18–25	16 (6.8%)
26–35	30 (12.8%)
36–45	66 (28.1%)
≥46	123 (52.3%)
Gender	
Male	96 (40.9%)
Female	139 (59.1%)
BMI (kg/m^2^)	34.09 ± 8.6
Marital status	
Single	43 (18.3%)
Married	149 (63.4%)
Divorced	25 (10.6%)
Widowed	18 (7.7%)
Monthly income (SR)	
<10,000	131 (55.7%)
10,000–25,000	89 (37.9%)
>25,000	15 (6.4%)
Highest educational level	
No formal education	12 (5.1%)
High school or lower	85 (36.2%)
Bachelor’s degree	113 (48.1%)
Master’s or PhD	25 (10.6%)
Duration of exercise per week	
Do not exercise	68 (28.9%)
<50 min	92 (39.1%)
50–100 min	38 (16.2%)
101–150 min	19 (8.1%)
>150 min	18 (7.7%)
Medical conditions *	
Hypertension (HTN)	80 (34%)
Kidney diseases	6 (2.6%)
CVS diseases	22 (9.4%)
None of the above	118 (50.2%)
Other	51 (21.7%)
Have you been diagnosed with Diabetes (DM)?	
No	94 (40%)
Yes	141 (60%)
How long have you been diagnosed with DM?	(*n* = 141)
<1 year	7 (5%)
1–5 years	34 (24.1%)
6–10 years	22 (15.6%)
>10 years	78 (55.3%)
How many times have you been hospitalized due to diabetes-related problems or complications in the past year?	(*n* = 141)
Never	105 (74.5%)
Once or twice	23 (16.3%)
3–5 times	8 (5.7%)
>5 times	5 (3.5%)
How many times have you visited the emergency department for diabetes-related problems in the past year?	(*n* = 141)
Never	98 (69.5%)
Once or twice	22 (15.6%)
3–5 times	12 (8.5%)
>5 times	9 (6.4%)
DM complications	(*n* = 141)
Retinopathy	30 (21.3%)
Neuropathy	37 (26.2%)
Kidney diseases	9 (6.4%)
None of the above	79 (56%)
Other	10 (7.1%)
Are you taking any diabetes medications?	(*n* = 141)
No	7 (5%)
Yes	134 (95%)
Are you taking Metformin and/or Sulfonylureas such as Glucophage or Daonil?	
No	133 (56.6%)
Yes, I only take metformin	59 (25.1%)
Yes, I only take sulfonylurea	13 (5.5%)
Yes, I take both	30 (12.8%)
How long have you been using GLP-1 agonists such as Saxenda, Ozempic, and Mounjaro?	
<1 year	123 (52.3%)
1–2 years	76 (32.3%)
>2 years	36 (15.3%)
Do you use diabetes medications for weight loss in addition to treating diabetes?	
No	121 (51.5%)
Yes	114 (48.5%)
Were you evaluated by a psychiatrist before starting to use GLP-1 medications such as Saxenda, Ozempic, or Mounjaro?	
No	160 (68.1%)
Yes	75 (31.9%)
Have you been diagnosed with any psychiatric disorders?	
No	200 (85.1%)
Yes	35 (14.9%)
Type of psychiatric disorder	(*n* = 35)
Mood disorders (depression or bipolar)	19 (54.3%)
Anxiety disorders (generalized anxiety disorder, social anxiety disorder, panic disorder/panic attacks)	17 (48.6%)
Eating disorders (such as bulimia nervosa, binge eating disorder)	1 (2.9%)
What type of psychiatric medication are you taking?	(*n* = 35)
Antidepressants/Anti-anxiety medications	30 (85.7%)
Antipsychotic medications	3 (8.6%)
I am not taking any psychiatric medications	3 (8.6%)
Were you diagnosed with a psychiatric illness before you started using GLP-1 medications such as Saxenda, Ozempic, or Mounjaro?	(*n* = 35)
No	9 (25.7%)
Yes	26 (74.3%)
Do any of your first-degree relatives (parents, siblings, children) have a history of psychiatric illness?	
No	205 (87.2%)
Yes	30 (12.8%)
Type of psychiatric illness	(*n* = 30)
Mood disorders (depression, bipolar disorder)	15 (50%)
Anxiety disorders (such as generalized anxiety disorder, social anxiety disorder, panic disorder/panic attacks)	6 (20%)
Psychotic disorders (such as schizophrenia)	8 (26.7%)
Other	6 (20%)

* Selecting multiple options is allowed; numerical data are presented as mean ± SD, and categorical data are presented as frequency (%).

**Table 2:** Participants’ responses to Generalized Anxiety Disorder-7 (GAD-7).

Item	Not at All	Several Days	More Than Half the Days	Nearly Everyday	Score
Over the last 2 weeks, how often have you been bothered by any of the following problems?					
Feeling nervous, anxious, or on edge	102 (43.4%)	87 (37%)	30 (12.8%)	16 (6.8%)	0.83 ± 0.9
Not being able to stop or control worrying	144 (61.3%)	57 (24.3%)	24 (10.2%)	10 (4.3%)	0.57 ± 0.84
Worrying too much about different things	117 (49.8%)	74 (31.5%)	31 (13.2%)	13 (5.5%)	0.74 ± 0.89
Trouble relaxing	99 (42.1%)	79 (33.6%)	41 (17.4%)	16 (6.8%)	0.89 ± 0.93
Being so restless that it is hard to sit still	159 (67.7%)	48 (20.4%)	22 (9.4%)	6 (2.6%)	0.47 ± 0.77
Becoming easily annoyed or irritable	97 (41.3%)	90 (38.3%)	34 (14.5%)	14 (6%)	0.85 ± 0.88
Feeling afraid as if something awful might happen	160 (68.1%)	50 (21.3%)	16 (6.8%)	9 (3.8%)	0.46 ± 0.79
Total score	4.82 ± 5				
No anxiety (0–4)	135 (57.4%)				
Mild anxiety (5–9)	60 (25.5%)				
Moderate anxiety (10–14)	29 (12.3%)				
Severe anxiety (15–21)	11 (4.7%)				
	Not difficult at all	Somewhat difficult	Very difficult	Extremely difficult	
How difficult have these made it for you to do your work, take care of things at home, or get along with other people?	133 (56.6%)	75 (31.9%)	24 (10.2%)	3 (1.3%)	

Numerical data are presented as mean ± SD, and categorical data are presented as frequency (%).

**Table 3:** Participants’ responses to Patient Health Questionnaire-9 (PHQ-9).

Item	Not at All	Several Days	More Than Half the Days	Nearly Everyday	Score
Over the last 2 weeks, how often have you been bothered by any of the following problems?					
Little interest or pleasure in doing things	130 (55.3%)	75 (31.9%)	24 (10.2%)	6 (2.6%)	0.6 ± 0.77
Feeling down, depressed, or hopeless	107 (45.5%)	94 (40%)	30 (12.8%)	4 (1.7%)	0.71 ± 0.75
Trouble falling or staying asleep, or sleeping too much	82 (34.9%)	81 (34.5%)	51 (21.7%)	21 (8.9%)	1.05 ± 0.96
Feeling tired or having little energy	59 (25.1%)	102 (43.4%)	48 (20.4%)	26 (11.1%)	1.17 ± 0.93
Poor appetite or overeating	56 (23.8%)	94 (40%)	60 (25.5%)	25 (10.6%)	1.23 ± 0.93
Feeling bad about yourself, or that you are a failure or have let yourself or your family down	161 (68.5%)	52 (22.1%)	13 (5.5%)	9 (3.8%)	0.45 ± 0.77
Trouble concentrating on things, such as reading the newspaper or watching television	156 (66.4%)	57 (24.3%)	17 (7.2%)	5 (2.1%)	0.45 ± 0.72
Moving or speaking so slowly that other people could have noticed. Or the opposite—being so fidgety or restless that you have been moving around a lot more than usual	174 (74%)	38 (16.2%)	18 (7.7%)	5 (2.1%)	0.38 ± 0.72
Thoughts that you would be better off dead, or off hurting yourself in some way	217 (92.3%)	14 (6%)	3 (1.3%)	1 (0.4%)	0.1 ± 0.37
Total score	6.13 ± 4.95				
No depression (0–4)	103 (43.8%)				
Mild depression (5–9)	75 (31.9%)				
Moderate depression (10–14)	38 (16.2%)				
Moderate-to-severe depression (15–19)	17 (7.2%)				
Severe depression (20–27)	2 (0.9%)				
Major depressive disorder	31 (13.2%)				
	Not difficult at all	Somewhat difficult	Very difficult	Extremely difficult	
How difficult have these made it for you to do your work, take care of things at home, or get along with other people?	134 (57%)	77 (32.8%)	22 (9.4%)	2 (0.9%)	

Numerical data are presented as mean ± SD, and categorical data are presented as frequency (%).

**Table 4:** Ordinal logistic regression analysis for factors associated with anxiety according to GAD-7 score.

Item	Univariate Analysis			Multivariable Analysis		
	Unadjusted OR	95%CI	*p*-Value	Adjusted OR	95%CI	*p*-Value
Age (years)						
18–25	Ref			Ref		
26–35	0.45	0.14–1.5	0.193	0.63	0.14–2.77	0.539
36–45	0.85	0.31–2.35	0.75	1.05	0.24–4.66	0.945
≥46	0.73	0.27–1.93	0.521	0.63	0.13–3.1	0.567
Gender						
Male	Ref			Ref		
Female	0.93	0.56–1.53	0.772	1.02	0.52–2	0.946
Marital status						
Single	Ref			Ref		
Married	1.2	0.61–2.37	0.599	1.53	0.52–4.53	0.441
Divorced	1.6	0.63–4.1	0.325	1.76	0.47–6.55	0.398
Widowed	1.27	0.41–3.9	0.677	0.95	0.2–4.6	0.95
Monthly income (SR)						
<10,000	Ref			Ref		
10,000–25,000	1.14	0.68–1.91	0.62	0.84	0.4–1.77	0.637
>25,000	0.48	0.15–1.54	0.216	0.28	0.05–1.46	0.131
Highest educational level						
No formal education	Ref			Ref		
High school or lower	0.33	0.1–1.12	0.076	0.55	0.13–2.29	0.416
Bachelor’s degree	0.49	0.15–1.62	0.244	0.94	0.2–4.31	0.936
Master’s or PhD	0.35	0.09–1.39	0.135	1.04	0.16–6.8	0.967
BMI (kg/m^2^)	1.01	0.99–1.04	0.313	1.01	0.97–1.04	0.679
Duration of exercise per week						
Do not exercise	Ref			Ref		
<50 min	0.71	0.39–1.29	0.256	0.52	0.25–1.07	0.075
50–100 min	1.07	0.5–2.27	0.866	1.1	0.46–2.65	0.833
101–150 min	0.32	0.11–0.94	0.039	0.25	0.07–0.9	0.035
>150 min	0.26	0.08–0.84	0.025	0.34	0.09–1.28	0.111
Medical conditions						
HTN	1.63	0.97–2.74	0.066	1.91	0.94–3.87	0.072
Kidney diseases	0.97	0.23–4.13	0.962	1.46	0.25–8.42	0.673
CVS diseases	0.94	0.38–2.33	0.897	0.58	0.2–1.68	0.319
DM	1.04	0.63–1.72	0.888	0.6	0.23–1.56	0.292
Taking Metformin and/or Sulfonylureas such as Glucophage or Daonil						
No	Ref			Ref		
Yes, I only take Metformin	1.13	0.62–2.06	0.687	1.29	0.5–3.31	0.594
Yes, I only take Sulfonylurea	0.7	0.21–2.39	0.574	1.2	0.27–5.32	0.812
Yes, I take both	1.6	0.77–3.36	0.211	1.57	0.54–4.56	0.411
Duration of use of GLP-1 agonists such as Saxenda, Ozempic, and Mounjaro						
<1	Ref			Ref		
1–2	1.35	0.78–2.35	0.29	1.41	0.73–2.7	0.304
>2	1.34	0.65–2.72	0.427	1.6	0.7–3.67	0.264
Using diabetes medications for weight loss in addition to treating diabetes	1.73	1.05–2.86	0.032	1.89	0.99–3.62	0.055
History of evaluation by a psychiatrist before starting to use GLP-1 medications such as Saxenda, Ozempic, or Mounjaro	1.84	1.09–3.09	0.022	0.89	0.47–1.68	0.723
History of diagnosis with any psychiatric disorders	9	4.51–17.96	<0.001	9.17	4.08–20.64	<0.001
Having first-degree relatives (parents, siblings, children) with a history of psychiatric illness	3.3	1.63–6.65	0.001	1.73	0.76–3.95	0.195

OR: odds ratio; CI: confidence interval; statistical significance at *p*-value < 0.05.

**Table 5:** Ordinal logistic regression analysis for factors associated with depression according to PHQ-9 score.

Item	Univariate Analysis			Multivariable Analysis		
	Unadjusted OR	95%CI	*p*-Value	Adjusted OR	95%CI	*p*-Value
Age (years)						
18–25	Ref			Ref		
26–35	0.39	0.12–1.24	0.11	0.49	0.12–1.89	0.298
36–45	0.67	0.24–1.88	0.448	0.98	0.24–4	0.976
≥46	0.6	0.22–1.6	0.306	0.7	0.15–3.18	0.64
Gender						
Male	Ref			Ref		
Female	0.93	0.58–1.5	0.771	1.3	0.69–2.43	0.415
Marital status						
Single	Ref			Ref		
Married	0.94	0.5–1.79	0.858	0.9	0.34–2.43	0.842
Divorced	1.51	0.6–3.8	0.378	1.14	0.34–3.79	0.832
Widowed	1.14	0.41–3.13	0.803	0.7	0.17–2.98	0.631
Monthly income (SR)						
<10,000	Ref			Ref		
10,000–25,000	1.22	0.74–2	0.431	0.97	0.48–1.94	0.924
>25,000	0.61	0.22–1.67	0.337	0.38	0.1–1.48	0.164
Highest educational level						
No formal education	Ref			Ref		
High school or lower	0.98	0.31–3.09	0.967	2.45	0.6–10.02	0.211
Bachelor’s degree	1.34	0.43–4.16	0.611	4.7	1.03–21.5	0.046
Master’s or PhD	1.2	0.33–4.36	0.783	6.91	1.18–40.44	0.032
BMI (kg/m^2^)	1.03	1–1.06	0.025	1.03	1–1.06	0.054
Duration of exercise per week						
Do not exercise	Ref			Ref		
<50 min	0.85	0.48–1.52	0.59	0.57	0.29–1.14	0.112
50–100 min	0.94	0.46–1.95	0.871	0.78	0.33–1.83	0.561
101–150 min	0.39	0.15–1.04	0.06	0.27	0.08–0.88	0.03
>150 min	0.39	0.15–1.02	0.054	0.4	0.13–1.27	0.121
Medical conditions						
HTN	1.75	1.06–2.88	0.028	1.77	0.91–3.44	0.09
Kidney diseases	1.07	0.27–4.3	0.921	1.88	0.36–9.8	0.452
CVS diseases	1.32	0.58–3	0.509	1.07	0.4–2.88	0.887
DM	1.27	0.79–2.05	0.327	0.86	0.36–2.07	0.733
Taking Metformin and/or Sulfonylureas such as Glucophage or Daonil						
No	Ref			Ref		
Yes, I only take metformin	1.3	0.74–2.29	0.357	1.06	0.44–2.53	0.897
Yes, I only take sulfonylurea	0.85	0.28–2.56	0.773	0.98	0.26–3.73	0.975
Yes, I take both	2.08	0.98–4.43	0.057	1.5	0.54–4.15	0.434
Duration of use of GLP-1 agonists such as Saxenda, Ozempic, and Mounjaro (year)						
<1	Ref			Ref		
1–2	0.89	0.52–1.53	0.673	0.79	0.42–1.46	0.445
>2	1.55	0.81–2.99	0.186	1.44	0.68–3.03	0.343
Using diabetes medications for weight loss in addition to treating diabetes	2.36	1.45–3.83	0.001	2.76	1.5–5.1	0.001
History of evaluation by a psychiatrist before starting to use GLP-1 medications such as Saxenda, Ozempic, or Mounjaro	1.78	1.07–2.96	0.026	0.97	0.53–1.79	0.926
History of diagnosis with any psychiatric disorders	9.04	4.36–18.74	<0.001	10.64	4.51–25.14	<0.001
Having first-degree relatives (parents, siblings, children) with a history of psychiatric illness	3.08	1.53–6.23	0.002	1.41	0.64–3.15	0.395

OR: Odds ratio, CI: Confidence interval; statistical significance at *p*-value < 0.05.

## Data Availability

Data from this study are available upon reasonable request directed to the corresponding author.
